# Pretreatment Epstein-Barr virus DNA in whole blood is a prognostic marker in peripheral T-cell lymphoma

**DOI:** 10.18632/oncotarget.21251

**Published:** 2017-09-23

**Authors:** Yu Ri Kim, Soo-Jeong Kim, June-Won Cheong, Haerim Chung, Ji Eun Jang, Yundeok Kim, Woo-Ick Yang, Yoo Hong Min, Jin Seok Kim

**Affiliations:** ^1^ Division of Hematology, Department of Internal Medicine, Gangnam Severance Hospital, Yonsei University College of Medicine, Seoul 06273, Republic of Korea; ^2^ Division of Hematology, Department of Internal Medicine, Severance Hospital, Yonsei University College of Medicine, Seoul 03722, Republic of Korea; ^3^ Department of Pathology, Severance Hospital, Yonsei University College of Medicine, Seoul 03722, Republic of Korea

**Keywords:** peripheral T-cell lymphoma, Epstein-Barr virus, whole blood, prognostic score

## Abstract

Because there are few studies regarding the clinical impact of circulating EBV-DNA in peripheral T-cell lymphomas (PTCLs), we tried to evaluate the role of EBV-DNA in whole blood as a prognostic factor for PTCL. We retrospectively reviewed 110 PTCL patients with median age of 63 (20-94) years. Forty-seven patients (42.7%) showed positive results for EBV-DNA, and these patients also had stage III/IV disease, elevated lactic dehydrogenase, and low albumin level (*P* = 0.007, *P* = 0.004, *P* = 0.002, respectively). The 5-year overall survival (OS) and progression free survival (PFS) were 21.0% and 18.0%. Univariable analysis showed that positive EBV-DNA was related with inferior OS and PFS (*P* = 0.015 and *P* < 0.001, respectively). Multivariable analysis showed that poor performance status, extranodal involvement more than one site and positive EBV-DNA results were related with OS and PFS (*P* < 0.001, *P* < 0.001, *P* = 0.007 and *P* = 0.001, *P* = 0.002, *P <* 0.001, respectively). Using these three variables, we made a new prognostic model which classified patients on risk as follows: low, no adverse factors; intermediate, 1 factor; or high, 2-3 factors. The new prognostic model could stratify the three groups for OS and PFS better than either international prognostic index or prognostic index of PTCL-u, and showed statistical significance in PTCL, not otherwise specified. This study suggests that whole blood EBV-DNA is related with aggressive clinical characteristics and inferior survival. The new prognostic model, which incorporates EBV-DNA, could better stratify PTCL patients.

## INTRODUCTION

Peripheral T-cell lymphoma (PTCL) is composed of 15-20% aggressive lymphoma and 5-10% non-Hodgkin's lymphomas (NHLs) and it is more prevalent in Asia than in Western countries [1]. PTCL is a group of heterogeneous disease with many different pathologic subtypes [2]. Although treatment outcome for B-cell NHL has been much improved, PTCL still has a dismal prognosis [3]. To predict the poor prognostic group of PTCL patients, the international prognostic index (IPI) had been widely used, as it has for other kinds of NHL [4]. However, IPI does not reflect the aggressive characteristics of PTCL, various kinds of scoring system such as prognostic index of PTCL-u (PIT) have been developed to predict survival in PTCL [5–7]. To improve the poor outcome of PTCL, it is important to stratify the broad spectrum of PTCL by defining the clinically usable prognostic factors.

Epstein-Barr virus (EBV) is a member of the γ-herpes virus family, which is found in many healthy adults [8]. EBV mainly resides in B-cells as harmless passenger, but it can infect B-cells or other cells, and could be a cause of malignant lymphoma [9]. Burkitt lymphoma, Hodgkin lymphoma (HL) and post-transplant lymphoproliferative disorder (PTLD) are known to be EBV-related diseases, although the mechanism of EBV is different according to each subtype [10]. Recently, many studies have reported on the role of circulating EBV-DNA in EBV-related lymphoma. Unlike EBV reactivation in immunocompromised patients, EBV-associated tumor, such as those found in HL and extranodal NK/T-cell lymphoma, nasal type (ENKL), contain fragmented EBV-DNA from tumor in immunocompetent patients. Plasma EBV-DNA in HL, as shown by real-time quantitative polymerase chain reaction (RQ-PCR), could be used as a biomarker to predict treatment response; moreover plasma EBV-DNA load has been associated with negative prognosis [11, 12]. Presence of EBV has been closely associated with development of ENKL, and high quantitative plasma EBV-DNA reflected the inferior overall survival (OS) in the patients with ENKL [13, 14]. Although plasma sample has been used in most studies, there is still controversy regarding which blood source could reflect the disease characteristics more accurately. Some studies have reported that whole blood EBV-DNA could also be a good source for predicting outcome in HL or diffuse large B-cell lymphoma (DLBCL) [15, 16]. Although EBV positivity in tumor tissue has been related with negative prognosis in PTCL, there are few data regarding the role of circulating EBV-DNA in PTCL [6, 17]. While serum EBV positivity has been related with poor progression-free survival (PFS) in PTCL, not otherwise specified (PTCL-NOS), it has not been shown to have clinical significance for predicting survival in multivariable analysis [18]. Moreover, quantitative analysis of EBV-DNA in the plasma has not been related with OS in PTCL [19].

In this study, we evaluated the presence of circulating EBV-DNA in whole blood from PTCL patients with respect to clinical outcome, and established a new prognostic model that incorporates EBV-DNA as a risk factor.

## RESULTS

### Baseline characteristics according to EBV-DNA

Median age of the 110 patients studied was 63 years (range, 20-94), and 65 (59.1%) were male patients. Thirty patients (27.3%) showed poor performance status, Eastern Cooperative Oncology Group (ECOG) ≥ 2. Ninety-seven patients (88.2%) were stage III/IV, and 37 patients (33.6%) had extranodal involvement more than one site. Seventy-five patients (68.2%) had elevated lactate dehydrogenase (LDH) level. Bone marrow involvement was detected in 49 patients (44.5%) among the 105 available patients. For IPI scores, 15 patients (13.6%) were classified as low risk, 28 (25.5%) as low-intermediate risk, 37 (33.6%) as high-intermediate risk, and 30 (27.3%) as high risk. For PIT scores among the 105 evaluable patients, 10 (9.5%) were assigned to group 1, 24 (22.9%) in group 2, 34 (32.4%) in group 3, and 37 (35.2%) in group 4. Eighty-nine patients (80.9%) could be evaluated for treatment response. Thirty patients (27.3%) achieved complete response (CR) and 28 (25.5%) achieved partial response (PR). Three patients (2.7%) had stable disease and 28 (25.5%) had progressed disease.

Forty-seven patients (42.7%) had positive results for EBV-DNA in whole blood. The copy number range was 5.4×10^2^ - 1.9×10^7^/mL and the median value of EBV-DNA was 1.73×10^4^/mL. The positive results for EBV-DNA had significantly associated with stage III/IV disease, elevated LDH level and low albumin level (*P* = 0.007, *P* = 0.004, *P* = 0.002, respectively). Other variables did not differ according to EBV-DNA positivity. Both EBV-encoded small RNA *in situ* hybridization (EBER-ISH) and EBV-DNA results were evaluable in 47 patients (39.4%); 10 patients (21.3%) had both positive results, and 16 patients (34.0%) had both negative results. Eleven patients (23.4%) were EBV-DNA positive but EBER negative, and 10 patients (21.3%) were EBV-DNA negative but EBER positive. There was no relation between EBV-DNA and EBER-ISH (*P* = 0.566). Patient characteristics according to EBV-DNA positivity are shown in Table 1. Treatment response did not differ according to EBV-DNA positivity (Table 1).

**Table 1:** Baseline characteristics according to Epstein-Barr virus results

	Total patientsn = 110 (%)	EBV-DNA positiven = 47 (%)	EBV-DNA negativen = 63 (%)	*P* value
Age				0.436
> 60 years	63 (57.3)	30 (63.8)	35 (55.6)	
≤ 60 years	47 (42.7)	17 (36.2)	28 (44.4)	
Sex				0.171
Male	65 (59.1)	24 (51.1)	41 (65.1)	
Female	45 (40.9)	23 (48.9)	22 (34.9)	
Performance status				0.198
ECOG 0-1	80 (72.7)	31 (34.0)	49 (77.8)	
ECOG ≥ 2	30 (27.3)	16 (66.0)	14 (22.2)	
Lymphoma subtype				0.775
PTCL, NOS	73 (66.4)	32 (68.1)	41 (65.1)	
AITL	22 (20.0)	10 (21.3)	12 (19.0)	
Others	15 (13.6)	5 (10.6)	10 (15.9)	
B symptoms				0.324
Negative	56 (53.8)	22 (47.8)	34 (58.6)	
Positive	48 (46.2)	24 (52.2)	24 (41.4)	
Stage				0.007
Stage I/II	13 (11.8)	1 (2.0)	12 (19.0)	
Stage III/IV	97 (88.2)	46 (98.0)	51 (81.0)	
Extranodal sites				0.999
0-1	73 (66.4)	31 (66.0)	42 (66.7)	
≥ 2	37 (33.6)	16 (34.0)	21 (33.3)	
Lactic dehydrogenase				0.004
Normal	35 (31.8)	8 (17.0)	27 (42.9)	
Elevated	75 (68.2)	39 (83.0)	36 (57.1)	
Bone marrow involvement				0.234
Negative	56 (53.3)	20 (45.5)	36 (59.0)	
Positive	49 (46.7)	24 (54.5)	25 (41.0)	
IPI at diagnosis				0.114
Low/Low-intermediate	43 (39.1)	14 (29.8)	29 (46.0)	
High-intermediate/High	67 (60.9)	33 (70.2)	34 (54.0)	
PIT at diagnosis				0.207
Group 1/2	34 (32.4)	11 (25.0)	23 (37.7)	
Group 3/4	71 (67.6)	35 (75.0)	38 (62.3)	
Response				0.247
CR/PR	58 (65.2)	18 (56.3)	40 (70.2)	
SD/PD	31 (34.8)	14 (43.8)	17 (29.8)	
ALC				0.557
≥ 1,000	66 (60.0)	30 (63.8)	36 (57.1)	
< 1,000	44 (40.0)	17 (38.2)	27 (42.9)	
Albumin				0.004
≥ 3.5	58 (52.7)	17 (36.2)	41 (65.1)	
< 3.5	52 (47.3)	30 (63.8)	22 (34.9)	
Ferritin				0.796
> 1,000	18 (17.5)	9 (19.1)	9 (16.1)	
≤ 1,000	85 (82.5)	38 (80.9)	47 (83.9)	
EBER-ISH				0.766
Negative	27 (58.7)	11 (55.0)	16 (61.5)	
Positive	19 (41.3)	9 (45.0)	10 (38.5)	

Abbreviations: EBV, Epstein-Barr virus; ECOG, Eastern Cooperative Oncology Group; PTCL, NOS, peripheral T-cell lymphoma, not otherwise specified; AITL, Angioimmunoblastic T-cell lymphoma; IPI, International Prognostic Index; PIT Prognostic Index for PTCL-u; CR, Complete response; PR, Partial response; SD, Stable disease; PD, progressive disease; ALC, Absolute lymphocyte count; EBER-ISH, EBV-encoded small RNAs-*in situ* hybridization.

### Influence of EBV-DNA on survival analysis

**Figure 1:**
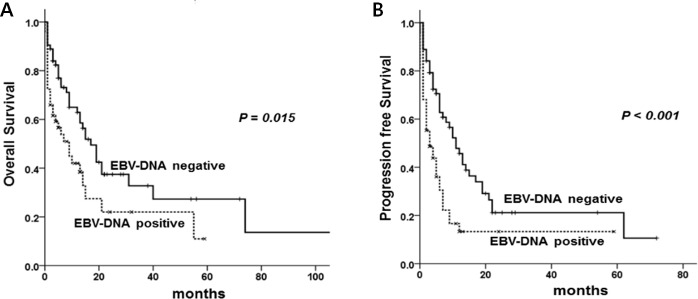
Overall survival **(A)** and progression free survival **(B)** according to EBV-DNA positivity.

The median follow-up period was 6.5 months (range, 0-137 months). Median OS and PFS were 14 months (95% confidence interval (CI), 9.8-18.1) and 6 months (95% CI, 3.6-8.3). Fiver-year OS and PFS were 21.0% and 18.0%, respectively. OS and PFS were significantly inferior in patients with the following factors; poor performance status (ECOG ≥ 2) (*P* < 0.001 and *P* < 0.001, respectively), extranodal involvement more than one site (*P* < 0.001, *P* = 0.003), and albumin < 3.5 g/dL (*P* = 0.007, *P* = 0.022). EBER-ISH results were not related with OS and PFS (*P* = 0.186, *P* = 0.980). OS and PFS of the patients with positive EBV-DNA were 9.0 months (95% CI, 3.6-14.3) and 3.0 months (95% CI, 0.7-5.2) while those of patients with negative EBV-DNA was 17.0 months (95% CI, 12.0–21.9) and 11.0 months (95% CI, 7.40-14.5) (*P* = 0.029 and *P* = 0.001, respectively) (Figure 1A, 1B).

In multivariable analysis, ECOG ≥ 2 (*P* < 0.001, *P* < 0.001), extranodal involvement more than one site (*P* < 0.001, *P* = 0.001) and positive EBV-DNA (*P* = 0.011, *P* = 0.001) were related with inferior OS and PFS (Table 2). The new scoring system incorporated these three factors, assigning one point to each factor (ECOG ≥ 2, extranodal involvement more than one site, positive EBV-DNA), and patients were classified as follows; low risk, no adverse factors; intermediate risk, presence of one factor; high risk, presence of two or more factors. As a result, 34 patients (30.9%) were designated as low risk, 47 (42.7%) as intermediate risk, and 29 (26.4%) patients as high-risk.

**Table 2:** Univariable and multivariable analyses for overall survival and progression-free survival

	OS		PFS	
	HR (95% CI)	*P* value	HR (95% CI)	*P* value
Univariable analysis				
Age > 60 years	1.12 (0.68-1.85)	0.651	0.91 (0.58-1.43)	0.688
Male sex	0.84 (0.51-1.37)	0.488	0.97 (0.62-1.53)	0.917
ECOG ≥ 2	3.03 (1.78-5.14)	< 0.001	2.59 (1.57-4.28)	< 0.001
Stage III/IV	1.61 (0.69-3.78)	0.266	2.20 (0.95-5.09)	0.065
Extranodal involvement > 1	2.46 (1.50-4.03)	< 0.001	2.01 (1.27-3.17)	0.003
Elevated LDH	1.82 (1.04-3.18)	0.034	1.39 (0.85-2.27)	0.183
Bone marrow involvement	1.56 (0.94-2.60)	0.083	1.34 (0.84-2.12)	0.214
Albumin < 3.5 g/dL	2.07 (1.26-3.40)	0.004	1.69 (1.07-2.65)	0.022
ALC < 1,000/μL	1.40 (0.84-2.32)	0.189	1.24 (0.78-1.96)	0.352
Ferritin > 1,000	1.36 (0.70-2.63)	0.355	1.41 (0.77-2.58)	0.257
EBER-ISH, positive	0.55 (0.20-1.48)	0.240	0.99 (0.46-2.09)	0.980
EBV-DNA, positive	1.79 (1.09-2.95)	0.021	2.05 (1.30-3.25)	0.002
Multivariable analysis				
ECOG ≥ 2	3.26 (1.89-5.64)	< 0.001	2.46 (1.48-4.08)	0.001
Extranodal involvement > 1	2.60 (1.58-4.29)	< 0.001	2.06 (1.30-3.26)	0.002
EBV-DNA, positive	2.01 (1.20-3.34)	0.007	2.27 (1.43-3.61)	< 0.001

Abbreviations: OS, overall survival; PFS, progression-free survival; HR, hazard ratio; CI, confidence interval; ECOG, Eastern Cooperative Oncology Group; LDH, lactic dehydrogenase; ALC, absolute lymphocyte count; EBER-ISH, EBV-encoded small RNAs-*in situ* hybridization; CR, complete response; EBV, Epstein-Barr virus.

### Comparison of three prognostic models

**Figure 2:**
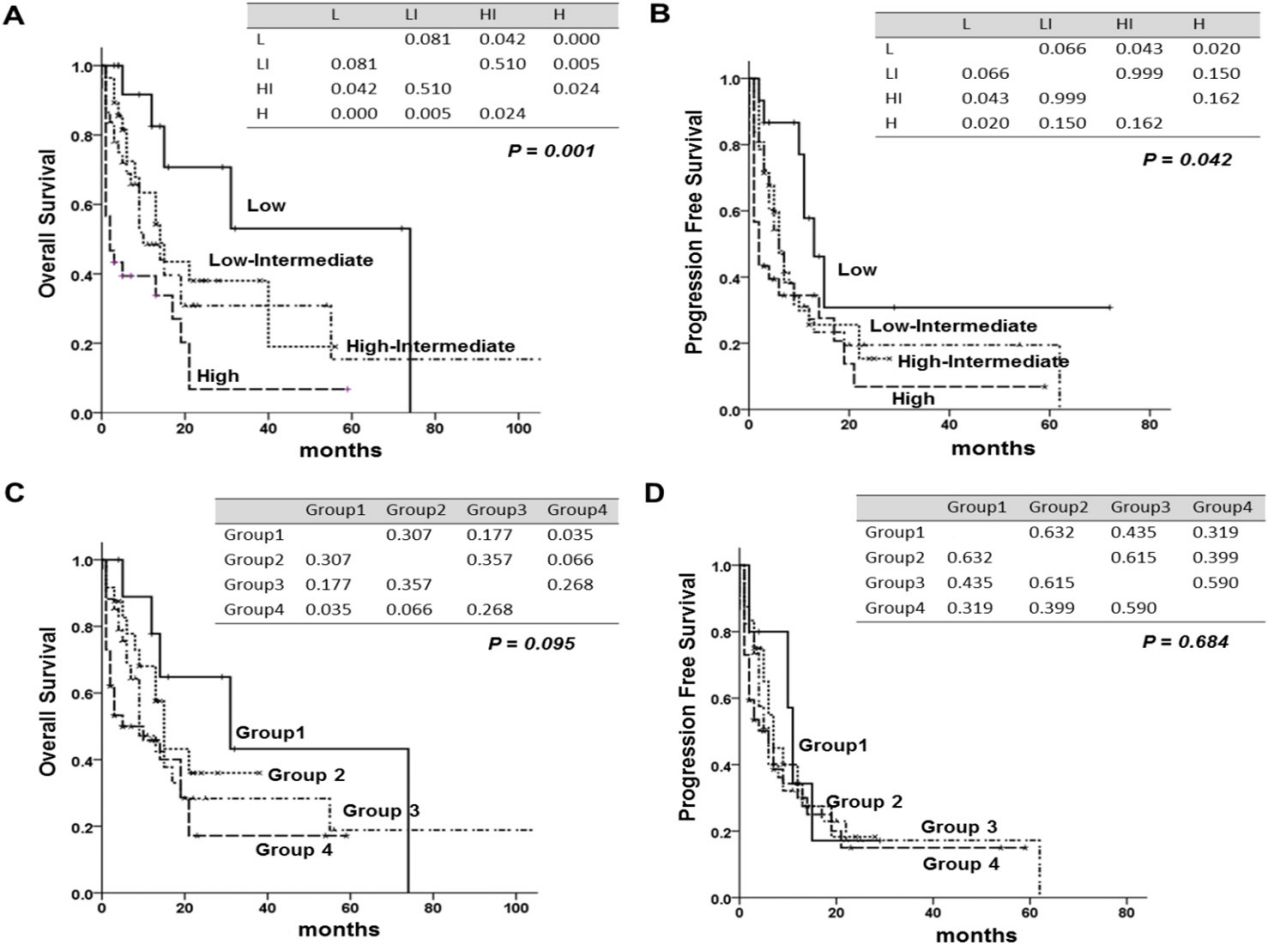
Overall survival (OS) and progression free survival (PFS) according to international prognostic index (IPI) and to prognostic index for PTCLu (PIT) **(A)** OS of IPI, **(B)** PFS of IPI **(C)** OS of PIT **(D)** PFS of PIT. The tables within the figure show the *P*-value between each factor by log-rank test. Abbreviations: L; Low, LI; low- intermediate, HI; high-intermediate, H; high.

**Figure 3:**
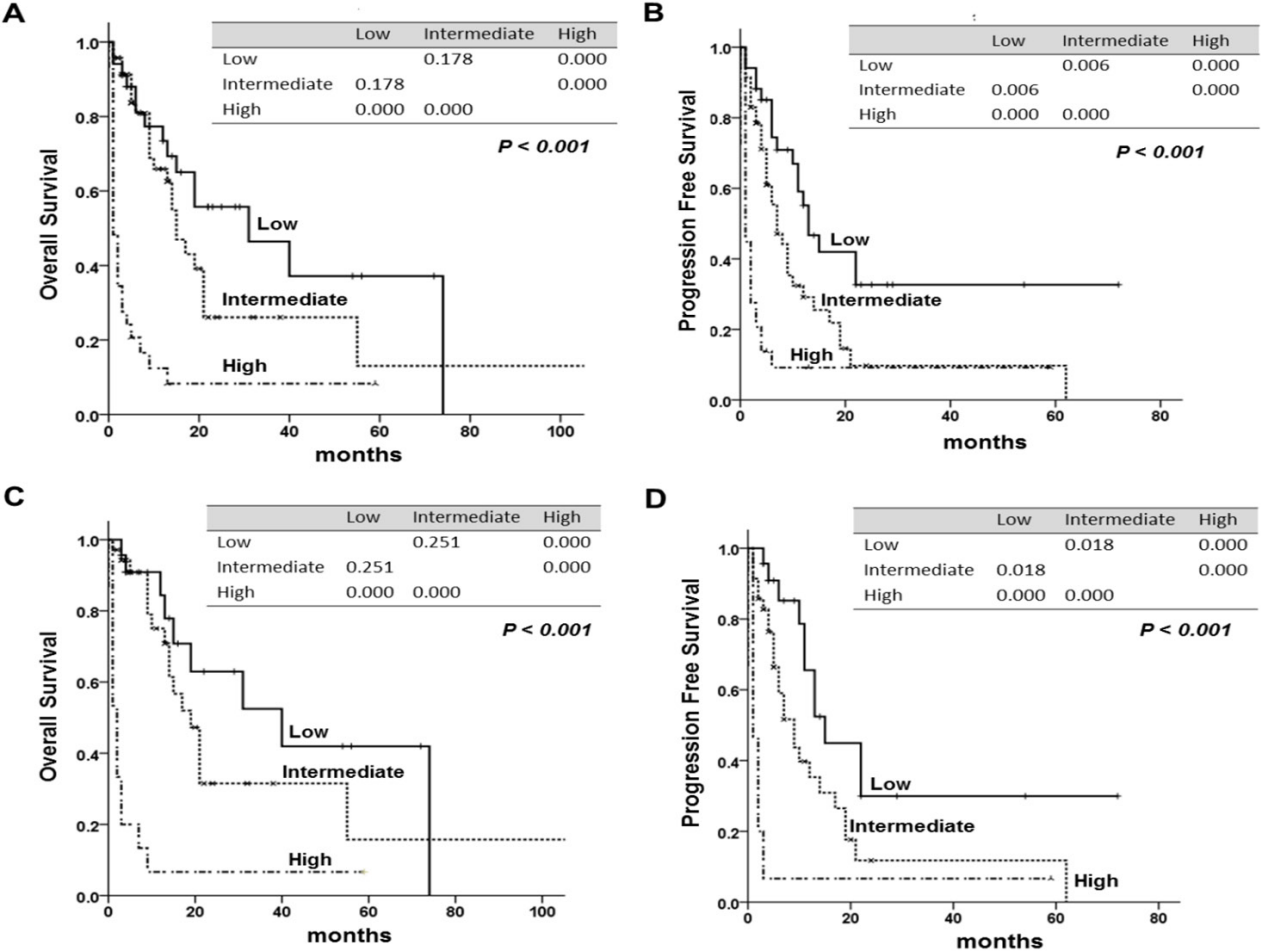
Overall survival (OS) and progression free survival (PFS) according to the new prognostic model. The tables within the figure show the comparison between each factor by log-rank test **(A)** OS, **(B)** PFS of the new prognostic model, **(C)** OS, **(D)** PFS of new prognostic model in peripheral T-cell lymphoma, NOS (PTCL, NOS). The tables within figure show the *P*-value between each factor by log-rank test.

We compared three prognostic scoring system, assessed by statistical method. Although IPI could identify OS and PFS for patients at all risk factor levels (*P* = 0.001, *P* = 0.042), it could not discriminate between low risk and low-intermediate risk (*P* = 0.081, *P* = 0.066), or between low-intermediate and high-intermediate risk patients (*P* = 0.051, *P* = 0.999) (Figure 2A, 2B). PIT could not discriminate the patients for OS and PFS for patients at all factor levels (*P* = 0.095, *P* = 0.684) (Figure 2C, 2D). Five-year OS and PFS of each prognostic model were shown in Table 3. The new prognostic model could identify different OS and PFS according to risk group (*P* < 0.001, *P* < 0.001) (Figure 3A, 3B), and it also showed statistical significance in the patients with PTCL, NOS (*P* < 0.001, *P* < 0.001) (Figure 3C, 3D). Akaike information criterion (AIC) value was lowest in the new prognostic model for predicting OS and PFS (AIC OS; 451.722, AIC PFS; 460.319) compared to IPI (AIC OS; 472.698, AIC PFS; 484.787) or PIT (AIC OS; 477.919, AIC PFS; 489.256) (Table 4). The new prognostic model showed better discrimination ability for OS and PFS than did either IPI or PIT, as shown by linear trend χ^2^ test (new prognostic model - linear trend χ^2^ test for OS; 24.63 and PFS; 24.17). Harrel's c index was calculated to evaluate predictive ability, and the new prognostic model showed the highest scores for predicting OS and PFS among the three prognostic scoring system.

**Table 3:** Comparison of survival between IPI, PIT and the new model

	N (%)	5-year OS(%)	*P* value	5-year PFS(%)	*P* value
**IPI**			0.001		0.042
Low risk	15 (13.6)	53.0		37.0	
Low-intermediate risk	28 (25.5)	23.0		17.0	
High-intermediate risk	37 (33.6)	18.0		17.0	
High risk	30 (27.3)	8.0		8.0	
**PIT**			0.095		0.684
Group 1	10 (9.5)	43.0		22.0	
Group 2	24 (22.9)	36.0		20.0	
Group 3	34 (32.4)	16.0		16.0	
Group 4	37 (35.2)	19.0		15.0	
**New model**			< 0.001		< 0.001
Low risk	34 (30.9)	41.0		33.0	
Intermediate risk	47 (42.7)	17.0		12.0	
High risk	29 (26.4)	7.0		9.0	

Abbreviations: IPI, International Prognostic Index; PIT, prognostic index for PTCLu; OS, overall survival; PFS, progression free survival.

**Table 4:** Comparison of prediction power between IPI, PIT and the new prognostic model

	IPI	PIT	New model
**OS**			
Goodness of fit (AIC)	472.698	477.919	451.722
Discriminatory ability			
(Linear Trend χ^2^ test)	10.55	6.19	24.63
Predictive ability			
(Harrell's c index)	0.667	0.638	0.715
**PFS**			
Goodness of fit (AIC)	484.787	489.256	460.319
Discriminatory ability			
(Linear Trend χ^2^ test)	3.46	1.54	24.17
Predictive ability			
(Harrell's c index)	0.601	0.577	0.702

Abbreviations: IPI, International Prognostic Index; PIT, prognostic index for PTCLu; OS, overall survival; PFS, progression free survival; AIC, Akaike information criterion.

## DISCUSSION

This study demonstrated that circulating EBV-DNA in whole blood were detected in about 40% of the newly diagnosed PTCL patients, and positive EBV-DNA results were related with aggressive clinical features and inferior OS and PFS. The new prognostic model, which was composed of three factors including positive circulating EBV-DNA, poor performance status (ECOG ≥ 2) and extranodal involvement more than one site, based on multivariable analysis, was shown to be more predictive for OS and PFS than was either IPI or PIT.

To date, the role of circulating EBV-DNA in lymphoma has been mostly evaluated in ENKL or HL. High EBV-DNA load in plasma was related with advanced stage and elevated LDH level, the patients with high EBV-DNA level were refractory to treatment and had inferior survival in ENKL [13, 14]. Plasma EBV-DNA has been recognized as an important prognostic factor to predict treatment response or survival in HL [11, 12]. On the other hand, the role of circulating EBV-DNA in PTCL has not been fully elucidated, although a few studies have reported the negative prognostic impact of EBER-ISH in T-cell lymphoma [17, 20, 21]. Based on gene expression profile data, EBER-ISH positive results in PTCL-NOS were related with fatal outcomes, and these patients expressed the immune response related gene [22]. Yang et al. showed that EBV-infected T-cells expressed higher levels of cytokine, IL-9, which suggested that EBV could affect the pathogenesis of EBV-associated T-cell disease [23]. Among PTCL, EBV infected B-cell clones were detected in AITL. These findings may distinguish AITL from other subtypes of PTCL [24, 25]. Therefore, EBV infection may play a role in development of PTCL. According to the recently revised WHO classification, node-based EBV+ PTCL was defined as nodal disease of PTCL-NOS with EBV positive in tumor cells [26, 27]. These findings suggested that EBV infection-associated PTCL could be considered as a distinct subgroup in PTCL. However, few studies have evaluated the role of EBV-DNA in PTCL. Two previous studies showed that positive EBV results in both serum and plasma were not related with clinical outcome in PTCL [18, 19]. However, those studies enrolled a small number of patients or used serum viral capsid antigen or early antigen tests. In this study, we tried to investigate the role of EBV-DNA in whole blood.

According to our data, EBV-DNA in whole blood was detected in 42.7% of PTCL patients and positive circulating EBV-DNA was related with advanced stage, elevated LDH level and low albumin level. Because stage and serum LDH level were related with tumor burden, positive whole blood EBV-DNA may be related with large tumor burden. In addition, low albumin level which was already known to be an important prognostic factor in PTCL [28, 29] showed a significant correlation with positive whole blood EBV-DNA. In our study, the proportion of patients with EBV-DNA positive results was not different according to age. This finding suggested that positive EBV-DNA in whole blood could not regarded as the abnormality associated only with age-related factor. For elderly patients with reduced the immune surveillance and vulnerability to EBV infection, it could be a cause of malignant lymphoma. However, we did not find a clear correlation between circulating EBV-DNA and age in PTCL patients. As in previous study, there was no clear relation between circulating EBV-DNA and EBER-ISH, and positive EBV-DNA results did not reflect the EBER status in tumor tissue [12, 15], thus circulating EBV-DNA is more predictive of clinical outcome than EBER status. In this study, the patients with positive EBV-DNA showed inferior OS and PFS as compared to those patients with negative EBV-DNA. Therefore, it is necessary to investigate the exact role of EBV for the progression of PTCL and develop EBV-specific therapeutic strategies for the PTCL patients with positive EBV-DNA.

We chose three variables showing significance based on multivariable analysis, including circulating EBV-DNA, performance status, and extranodal involvement more than one site, and we suggested the new prognostic model. The new scoring system showed the most statistical significance as compared to IPI or PIT. Although IPI has been widely used like other kinds of NHLs, it could not reflect the aggressive clinical characteristics of PTCL, thus PIT including bone marrow involvement was suggested [7]. However, IPI could not differentiate the patients between the low and low-intermediate, or between low-intermediate and high-intermediate risk group for OS and PFS. PIT also could not identify each group to different outcome in our cohort. On the contrary, the new prognostic model incorporating EBV-DNA results could stratify the PTCL patients well, and it also showed statistical significance for the patents with PTCL, NOS. The new prognostic model showed the lowest AIC, it indicated a more suitable to predict the OS and PFS, and the highest linear trend χ^2^ test and Harrell's c index among the three models, suggesting that it has the best discriminatory ability and predictive accuracy [30, 31].

There has been still some argument about the best blood source for detecting EBV-DNA. Most studies emphasized that pretreatment EBV-DNA in plasma is related with treatment response and survival, because plasma EBV DNA reflects the tumor burden [12, 13]. However, it has a short half-life with several minutes, and the samples are hard to handle. In contrast, EBV quantitation in whole blood can detect EBV-DNA presented in both the cellular component and cell-free compartment. The EBV-DNA level in whole blood is usually higher than in plasma. Therefore, EBV-DNA assay using whole blood is relatively easy to detect and easy to handle [32, 33]. Recent studies showed that EBV-DNA in whole blood was useful for predicting the clinical outcome in HL or DLBCL [15, 16]. In relapsed/refractory ENKL, EBV-DNA in whole blood is a more sensitive marker than plasma EBV-DNA in predicting response or adverse events of SMILE (steroid, methotrexate, ifosfamide, L-asparaginase, and etoposide) chemotherapy [34]. Although we did not detect the circulating tumor cells in this study, it was possible to detect circulating tumor cells in patients with bone marrow involvement. In this study, proportion of the patients with bone marrow involvement was not different according to the positivity of EBV-DNA. Therefore, it is not possible to conclude that EBV-DNA in whole blood is only related with circulating tumor cells. Similarly, previous study reported that the ENKL patients with EBV-DNA positive in whole blood did not related with the leukemic presentation [34]. To reveal the exact mechanism of detecting EBV-DNA in whole blood, it is necessary to analyze the DNA fragment lengths, and compare the EBV-DNA level between whole blood and plasma. We evaluated all PTCL patients with immunocompetent status to exclude the tumorigenesis rising from EBV reactivation in patients with immunocompromised status such as PTLD or human immunodeficiency virus (HIV)-associated lymphoma. Although there are concerns that the detection of EBV-DNA using whole blood could not discriminate the viral load from tumor cell or latently infected benign B-cells [35], EBV-DNA level in whole blood might reflect actual clinical situations, because it includes both cellular and plasma component [33, 36].

This study has some limitations. First, it was composed of many heterogeneous subgroups of PTCL patients and performed in single center, retrospectively. To improve these limitations, we included the all consecutive patients who were diagnosed with PTCL in our institution. Second, both EBER-ISH and EBV-DNA results were evaluable in only 47 patients (39.4%), because EBER-ISH was not a test routinely given upon diagnosis of PTCL, thus our results did not exactly reflect the association between EBER-ISH and circulating EBV-DNA in whole blood. However, this study did provide statistically powerful values using AIC, linear trend χ^2^ test and Harrell's c index. To reveal the clinical impact and meaning of EBV-DNA in PTCL, it will be needed to validate data in a large population prospective study, with monitoring of serial follow ups.

This study suggests that patients with pretreatment positive EBV-DNA results in whole blood are related with aggressive clinical features and inferior OS and PFS. The new prognostic model composed of positive EBV-DNA, poor performance status, and extranodal involvement more than one site, is more suitable for predicting outcomes for PTCL than are either IPI or PIT.

## MATERIALS AND METHODS

### Patients

Between January 2002 and December 2015, 2,409 patients were newly diagnosed with lymphoma at Severance Hospital, Yonsei University College of Medicine, Seoul, Korea. Among these patients, we retrospectively evaluated the 259 (10.8%) patients who were diagnosed PTCL. Histology was confirmed by hematopathology specialists. We excluded 85 patients who had different clinical characteristics compared to PTCL; ENKL, primary cutaneous T-cell lymphoma, anaplastic large-cell lymphoma (ALCL)-anaplastic lymphoma kinase (ALK) positive, precursor T-lymphoblastic leukemia/lymphoma, HIV-associated lymphoma or PTLD.

In total, 110 PTCL patients were included in this study, 73 (66.4%) with PTCL, NOS, 22 (20.0%) with angioimmunoblastic T-cell lymphoma, 12 (10.9%) with ALCL-ALK negative, and 3 (2.7%) with enteropathy-associated T-cell lymphoma. All patients had whole blood EBV-DNA results before starting chemotherapy. One hundred patients (90.9%) were treated with first line chemotherapy while the other 10 patients could not receive chemotherapy due to poor performance status or infection (Supplementary Table 1). Response to treatment was assessment by using international workshop response criteria [37, 38]. We excluded patients who received upfront autologous hematopoietic stem cell transplantation (ASCT) because upfront ASCT could be a standard therapeutic option only for young and chemo-sensitive patients and upfront ASCT may improve the treatment outcomes.

### Prognostic index

IPI scores were based on age, ECOG performance status, LDH level, the number of extranodal sites involvement and Ann Arbor stage [4]. Four risk groups were defined based on IPI scores: 0 to 1, low risk; 2, low-intermediate risk; 3, high-intermediate risk; and 4 to 5, high risk. PIT was scored by age, ECOG performance status, LDH level, and bone marrow involvement. The four risk groups were defined by PIT scores: 0, group 1; 1, group 2; 2, group 3; and 3 to 4, group 4 [7].

### RQ-PCR of EBV-DNA

EBV-DNA was isolated from whole blood samples by manual extraction using QIAamp DNA Blood Mini Kits (QIAGEN Inc., Valencia, CA, USA). Quantitative real-time PCR (RealArt EBV LC PCR kit, Qiagen, Hamburg, Germany) was performed using a LightCycler 2.0 (Roche Molecular Diagnostics, Pleasanton, CA, USA). Thermal cycling was initiated at 50°C for 1 minute, followed by a first denaturation step of 95°C for 10 minutes, and then 45 cycles of 95°C for 15 seconds and 50°C for 1 minute. Results are expressed in copies/ml of total EBV-DNA calculated using a standard curve. The lowest detection limit of EBV-DNA in whole blood was 510 copies/mL.

### *In situ* hybridization for EBV

ISH analysis of EBER was performed for detection of latent EBV infection in paraffin-embedded tissue sections, according to the supplier's instructions (Novocastra, Newcastle upon Tyne, United Kingdom). Paraffin sections were dewaxed in xylene and hydrated through graded alcohols and distilled water. The slides were then treated with proteinase K (10 μg/mL, Dako) at 37°C for 15 minutes and washed in Tris-buffered saline (50 mmol/L Tris-HCl, 150 mmol/L NaCl; pH = 7.6) containing 0.1% Triton X-100. The slides were incubated with fluorescein-conjugated EBER probes (Novocastra) while covered with cover glasses at 37°C for 120 minutes. The slides were then washed in Tris-buffered saline followed by a washing with a stringent wash solution at 45°C for 20 minutes. After being washed, the slides were incubated with an alkaline phosphatase-conjugated antifluorescein isothiocyanate antibody (Novocastra) for 30 minutes, and then washed in Tris-buffered saline. Next, signals were detected by using a BCIP/NBT chromogen kit (Dako), and the sections were counterstained with nuclear fast red. Positive and negative control slides were processed in parallel.

### Statistical analysis

The statistical significance of categorical variables was examined using Fisher's exact tests. Survival curves were estimated using the Kaplan-Meier test and survival difference was evaluated using the log-rank test. OS was measured from the date of diagnosis until death from any cause, and surviving patients were censored at the last follow-up date. PFS was defined from the date of diagnosis to the date of disease progression, relapse, or death from any cause. To rule out multicollinearity between the included parameters, variance inflation factor (VIF) was assessed. VIF values were checked for the independent variables included in the multivariable model. VIF values ranged from 1.08 to 1.35, indicating the absence of multicollinearity (Supplementary Table 2).

We calculated the AIC, for each prognostic score to demonstrate which score was more explanatory and informative in predicting survival. The AIC is a commonly used measure for comparing competing models, and a smaller AIC indicates the preferred model [30]. Lastly, the time-dependent receiver operating characteristic curve method was used to compare the three prognostic models on predictive accuracy for OS or PFS over the entire range of follow-up times [31]. A *P*-value < 0.05 was considered statistically significant for all analyses. All statistical analyses were performed using SPSS for Windows, version 20.0 (IBM Corp., Armonk, NY, USA).

## SUPPLEMENTARY MATERIALS TABLES


