# Supplementary material for: Pretreatment Epstein-Barr virus DNA in whole blood is a prognostic marker in peripheral T-cell lymphoma
Source: Oncotarget. 2017 Sep 23;8(54):92312–23. doi: 10.18632/oncotarget.21251 (PMC5696183; doi:10.18632/oncotarget.21251)
Supplement: Supplementary file 1 [file oncotarget-08-92312-s001.pdf]

Pretreatment Epstein-Barr virus DNA in whole blood is a prognostic marker in peripheral T-cell lymphoma

SUPPLEMENTARY MATERIALS

Supplementary Table 1: Frontline chemotherapy regimens and number of cycles

	n (%)	Median cycle (Range)
CHOP-based chemotherapy	78 (78.0%)	6 (1-8)
IMEP-based chemotherapy	19 (19.0%)	4 (1-6)
Other regimens	3 (3.0%)	2 (1-3)

Abbreviations: CHOP, cyclophosphamide, doxorubicin, vincristine, and prednisone; IMEP, ifosfamide, methotrexate, etoposide, and prednisone.

Supplementary Table 2: Variance inflation factor of each prognostic factors

	VIF
ECOG ≥ 2	1.200
EBV-DNA, positive	1.172
Stage III/IV	1.262
Albumin < 3.5 g/dL	1.346
Extranodal involvement > 1	1.079
Elevated LDH	1.181

Abbreviations: VIF, variance inflation factor; ECOG, Eastern Cooperative Oncology Group; EBV, Epstein-Barr virus, LDH, lactic dehydrogenase.
